# Virus-like particle vaccines with epitopes from porcine epidemic virus and transmissible gastroenteritis virus incorporated into self-assembling ADDomer platform provide clinical immune responses in piglets

**DOI:** 10.3389/fimmu.2023.1251001

**Published:** 2023-10-24

**Authors:** Pengfei Du, Quanhui Yan, Xiao-Ai Zhang, Weijun Zeng, Kaiyuan Xie, Zhongmao Yuan, Xiaodi Liu, Xueyi Liu, Lihong Zhang, Keke Wu, Xiaowen Li, Shuangqi Fan, Mingqiu Zhao, Jinding Chen

**Affiliations:** ^1^ Department of Preventive Veterinary Medicine, College of Veterinary Medicine, South China Agricultural University, Guangzhou, Guangdong, China; ^2^ Agro-Biological Gene Research Center, Guangdong Academy of Agricultural Sciences, State Key Laboratory of Livestock and Poultry Breeding, Guangzhou, China

**Keywords:** porcine epidemic diarrhea virus, transmissible gastroenteritis virus, virus-like particles, ADDomer, insect baculovirus expression system, epitope

## Abstract

**Introduction:**

Porcine epidemic diarrhea virus (PEDV) and transmissible gastroenteritis virus (TGEV) are major intestinal coronaviruses that cause vomiting, diarrhea, dehydration, and mortality in piglets. These viruses coexist and lead to significant economic losses in the swine industry. Virus-like particles (VLPs) have emerged as promising alternatives to conventional inactivated vaccines due to their exceptional safety, efficacy, and ability to provide multi-disease protection with a single dose.

**Methods:**

Our study focused on specific antigenic epitopes from the PEDV S protein (SS2 and 2C10 regions) and the TGEV S protein (A and D sites) as target candidates. These epitopes were integrated into the ADDomer framework, and we successfully generated recombinant proteins AD, AD-P, AD-T, and AD-PT using the baculovirus expression vector system (BEVS). By meticulously optimizing conditions in High Five cells, we successfully expressed and purified the recombinant proteins. Subsequently, we developed the recombinant ADDomer-VLP vaccine and conducted a comprehensive evaluation of its efficacy in piglets.

**Results:**

Following ultrafiltration concentration and sucrose gradient centrifugation purification, the recombinant proteins self-assembled into VLPs as observed by transmission electron microscopy (TEM). Administration of the vaccine did not result in any adverse reactions in the immunized piglets. Additionally, no significant instances of fever were detected in any of the experimental groups, and there were no notable changes in average daily weight gain compared to the control group that received PBS. The recombinant ADDomer-VLP vaccines demonstrated strong immunogenicity, effectively stimulating the production of neutralizing antibodies against both PEDV and TGEV. Moreover, the recombinant ADDomer-VLP vaccine induced elevated levels of IFN-γ, IL-2, and IL-4, and enhanced cytotoxic T lymphocyte (CTL) activity in the peripheral blood of piglets.

**Discussion:**

These recombinant VLPs have demonstrated the ability to induce strong cellular and humoral immune responses in piglets, making them an incredibly promising platform for the rapid and simplified development of epitope vaccines.

## 1 Introduction

Porcine epidemic diarrhea virus (PEDV) and Transmissible gastroenteritis virus (TGEV), which belong to the family of Swine enteric coronaviruses (SeCoV) are responsible for causing Porcine epidemic diarrhea (PED) and Transmissible gastroenteritis (TGE) in pigs (1). Pigs of all age groups are susceptible to infection by PEDV and TGEV, with pregnant sows capable of transmitting the viruses to newborn piglets through vertical transmission. In cases of infection among lactating piglets, the mortality rate can reach alarming levels of up to 100% (2). The primary mode of transmission for PEDV is through the fecal-oral route. However, recent studies have indicated the potential for airborne transmission of PEDV as an additional route of spread (3). The PEDV strain has rapidly spread to various countries across Asia, North America, and Europe (4–12). Particularly noteworthy is the significant prevalence of GII PEDV in China since 2010, resulting in a surge in morbidity and mortality rates among afflicted piglets (8). In 1946, the initial documentation of TGEV occurred in the United States (13), followed by subsequent identifications in Europe, Asia, Africa, and South America (5, 14–16). This widespread distribution of TGEV has resulted in substantial economic losses within the global pig industry. The primary modes of TGEV transmission include fecal-oral transmission, respiratory transmission, and breastfeeding transmission (17, 18). TGEV and PEDV often present clinically mixed infections with indistinguishable symptoms and similar pathological changes. However, cross-protection between the two viruses is limited (19).

The widely used PEDV/TGEV dual vaccines are mainly inactivated and weakly virulent vaccines prepared from isolated strains. In March 2015, a trivalent vaccine developed from attenuated TEGV (strain H), PEDV (strain CV777, subgroup GI-a) and porcine rotavirus (strain NX) was approved in China. Sows vaccinated with inactivated or attenuated vaccines will produce sIgA and IgG antibodies in colostrum, which will allow piglets to establish passive immunity to PEDV and TGEV. Existing commercial vaccines still have several drawbacks. Weak vaccines carry the risk of reverting to strong virulence, and there is also the possibility of antigenic mutation in the virulent strain. Inactivated vaccines can experience changes in immunogenicity during the inactivation process, often necessitating multiple doses and booster injections. Additionally, piglets often do not receive sufficient protective antibodies through the maternal route from immunized sows (20, 21).

VLPs are empty structures formed by assembling viral proteins without any nucleic acids present inside. However, they maintain numerous essential characteristics of the viral capsid, such as precise structural and size uniformity, biocompatibility, stability, immunogenicity, and affinity for cells (22). The formation of VLP shells occurs through a spontaneous process, wherein the interactions among protein monomers contribute to the highly organized structure. These unique properties of VLPs make them appealing nanoplatforms for various applications, as they can effectively showcase and accommodate functional biomolecules.

ADDomer is an adenovirus-derived self-assembling nanoparticle scaffold based on multimeric proteins. It enables plug-and-play access to multiple immunogenic epitopes of pathogens (23). Every ADDomer VLP particle is formed through the assembly of 12 pentameric protein complexes, each measuring 300 kDa in size. These particles offer up to 360 insertion regions for the display of antigenic epitopes. Remarkably, they demonstrate outstanding thermal stability comparable to that of conventional VLPs. This breakthrough in the field of traditional vaccines overcomes significant obstacles, simplifying the vaccine design and production process to a great extent. In previous preclinical trials, a vaccine utilizing ADDomer to showcase the chikungunya virus E2 protein demonstrated promising immunogenicity (23). In the context of the SARS-CoV-2 pandemic, one study targeted modifications to the ADDomer platform and constructed an ADDomer VLP that expresses the RBD of the SARS-CoV-2 S-protein. Immunization of mice showed that the VLP triggered a significant humoral immune response in the body, and neutralization assays with a SARS-CoV-2 S-protein pseudovirus demonstrated a very high serum neutralization potency after immunization (24). ADDomer, with its BEVS-based plug-and-play antigen display platform, holds great promise as an innovative VLP vaccine vector against emerging pathogens.

PEDV and TEGV S proteins can induce the body to produce neutralizing antibodies (25). In the PEDV S protein, four B-cell epitopes have been identified. These include the core neutralizing epitope (COE) located in the S1 region (aa 499-638), SS2 (aa 748-755), SS6 (aa 764-771), and 2C10 (aa 1368-1374) in the S2 region (26). The SS2 epitope (aa 748-755) and epitope 2C10 (aa 1368-1374) have demonstrated the ability to induce neutralizing antibodies against PEDV. Importantly, these epitopes have been found to be conserved in all wild strains of PEDV isolated from China (27, 28). However, it is worth noting that the COE epitope region and the SS6 epitope exhibit significant diversity among the majority of wild PEDV strains, serving as high-frequency mutation regions within the epitope region (29). Particularly in the CT-P120 and PT-P96 strains, epitope region mutations may impair neutralizing antibody recognition, and the F636R and F636S mutations in the COE epitope region may lessen the responsiveness of viral neutralizing antibodies (30). Studies have demonstrated that both the SS2 and 2C10 epitopes are capable of inducing PEDV-specific neutralizing antibodies in mice (31, 32). Consequently, the SS2 and 2C10 epitope regions serve as valuable references for the development of antigenic epitope vaccines against PEDV.

Based on the protease hydrolysis sites, TGEV S proteins can be split into the S1 region (aa 1-790) and the S2 region (aa 790-1383) (33). The S1 region is distinguished as S1-NTD and S1-CTD and contains RBD (aa 560-655). The epitopes crucial for stimulating neutralizing antibodies in the TGEV S protein are situated in the NTD of the S1 protein. Earlier studies have identified four antigenic sites within the anterior portion of the S1 region, namely C (aa 49-52), B (aa 75-142), D (aa 385-386), and A (aa 540-592) (34, 35). The A and D sites, with the potential presence of multiple RBDs on their surface, play a vital role in the production of neutralizing antibodies (36). The A site, located on the surface of the TGEV virus particle, is characterized by the presence of crucial amino acids 538, 543, and 591. These amino acids are essential for maintaining the proper conformation of the site. The D site, a highly conserved linear antigenic site, shares similarities with the A site in its ability to stimulate the production of neutralizing antibodies within the body (37, 38). Consequently, the A and D sites of the S protein serve as crucial target antigenic regions for the prevention and control of TGEV through the utilization of novel vectors.

Therefore, the SS2 and 2C10 regions of PEDV S protein and the A and D sites of TGEV S protein were selected as candidate antigenic epitopes in this study. These epitopes were inserted into the adomer framework and the recombinant proteins AD, AD-P, AD-T, and AD-PT were expressed by BEVS.Subsequently, the self-assembled recombinant ADDomer-VLPs were prepared into a vaccine, and the immune efficacy of the recombinant ADDomer-VLPs vaccine was evaluated by immunization experiments in piglets.

## 2 Materials and methods

### 2.1 Cells culture

Vero cells, ST cells, DH10Multibac receptor cells, and transfer plasmid pFBDM were obtained from the Department of Veterinary Microbiology and Immunology, South China Agricultural University. sf9 cells and High Five cells were purchased from Beijing Yiqiao Shenzhou Technology Co. Vero cells and ST cells were cultured using Dulbecco’s modified Eagle’s medium (Gibco, USA) containing 10% fetal bovine serum (Gibco, USA) in a 37°C, 5% -CO2 incubator. sf9 cells and High Five cells were cultured using SIM SF medium and SIM High Five medium purchased from Beijing Yiqiao Shenzhou Technology Co., Ltd. at 27°C and 110 rpm in a shaker.

### 2.2 Protein design

The PEDV AJ1102 strain (GenBank Accession: JX188454.1) and the TGEV SHXB strain (GenBank Accession: KP202848.1) available in GenBank were used as references. From these strains, the SS2 and 2C10 antigenic regions of the PEDV S protein and the A and D antigenic sites of the TGEV S protein were selected as exogenous antigens for further investigation (**Table 1**). The ADDomer recombinant protein AD, as well as the recombinant proteins AD-P, AD-T, and AD-PT, were generated by incorporating different tandem forms of exogenous antigens into specific regions of the ADDomer structure. Specifically, the PEDV antigen epitope was inserted into the VL region, RGD1 region, and RGD2 region of ADDomer. The sequences provided in Patent No. US2020325179A1 were utilized as references for the design and construction of these recombinant proteins (39). For the spatial structure simulation of the recombinant VLP, the crystal structure file with the PDB number 6hcr was chosen. The simulation was conducted using UCSF Chimera X, a software tool commonly used for visualizing and analyzing molecular structures. The designed exogenous gene sequences were subjected to optimization for insect cell-preferred codons.

**Table 1:** Selected S protein antigen sites.

Virus	Name	Amino acid sites	Amino acid sequence
PEDV	SS2	aa 748～755	YSNIGVCK
	2C10	aa 1368～1374	GPRLQPY
TGEV	SA	aa 538～591	KRSGYGQPIASTLSNITLPMQDHNTDVYCIRSDQFSVYVHSTCKSALWDNIFKR
	SD	aa 373～398	CYTVSDSSFFSYGEIPFGVTDGPRYC

### 2.3 Virus acquisition

Subsequently, the optimized gene sequences were synthesized and directly inserted into the pFBDM plasmid by Sangon (Sangon, China). This process resulted in the generation of recombinant transfer plasmids, namely pFBDM-AD, pFBDM-AD-P, pFBDM-AD-T, and pFBDM-AD-P & TGEV.

The recombinant transfer plasmids were subjected to agarose gel electrophoresis for identification, followed by submission to Sangon (Sangon, China) for sequencing analysis. The constructed recombinant transfer plasmid was transformed into DH10Multibac receptor cells, and then the monoclonal colonies were picked and incubated on shaker at 37°C for 6 h. 150 µL of bacterial solution was aspirated and spread on LB agar medium containing Gen, Kan, Amp, IPTG, and X-Gal, and the blue and white spots of the colonies were observed in an incubator after the dishes were placed upside down in the incubator for 48 h. The colonies were then placed in the incubator for 48 h to observe the blue and white spots. White monoclonal colonies were picked and cultured for 24 h at 37°C on a shaker, followed by extraction of recombinant baculovirus plasmids The recombinant baculovirus plasmid successfully identified by PCR was transfected into sf9 cells, which were subsequently incubated at 27°C for 96 h in an incubator (40). The recombinant baculoviruses obtained by centrifugation of the culture medium and aspiration of the supernatant. They were named Ac-AD, Ac-AD-P, Ac-AD-T and Ac-AD-PT, respectively.

The titer of P1 generation viruses is relatively low, so P1 generation recombinant baculoviruses can be passaged in suspension culture of sf9 cells to increase the titer. We inoculated the recombinant baculovirus into sf9 cells in a shaker at 27°C and cultured at 140 r/min for 96 h, and passaged to the P3 generation of recombinant baculovirus.

### 2.4 Virus titer determination

The AceQ Universal SYBR qPCR Master Mix (Vazyme, USA) was utilized for absolute quantitative RT-qPCR analysis. Multiple dilutions of known concentrations of plasmid pMD18-T-Ac were performed and a standard curve was plotted based on the copy number and Cq value of the plasmid. Recombinant baculovirus DNA was extracted using the Omega Viral DNA Kit (Omega Bio-Tek, USA) following the provided instructions, and this DNA served as the template for the RT-qPCR assay. The Cq value obtained from each sample well was then used to determine the recombinant baculovirus nucleic acid copy number by interpolating it into the standard curve. Finally, the virus titer was calculated using the formula specified in the user manual of the Bac-to-Bac^®^ Baculovirus Expression System from Invitrogen.

### 2.5 Protein identification

Cell precipitates from the P1 to P3 generations were collected separately for protein expression verification through Western blot analysis. The recombinant proteins were detected using specific primary antibodies. The mouse-derived anti-ADDomer polyclonal antibody, mouse-derived anti-PEDV S1 protein monoclonal antibody (Guangzhou Qianxun Biological Co., Ltd., China), and rabbit-derived anti-TGEV S1 protein polyclonal antibody (Alpha Diagnostic International, USA) were used as primary antibodies for detection. Subsequently, secondary antibodies such as goat anti-mouse IgG-HRP antibody or goat anti-rabbit IgG-HRP antibody (Shanghai Biyuntian Biotechnology Co., Ltd., China) were employed. Primary antibodies were incubated overnight at 4°C and secondary antibodies at room temperature for 1h.The displayed images were obtained using an ECL chemiluminescent solution (Shanghai Yase Biotechnology Co., Ltd., China) on PVDF membranes.

The recombinant proteins were detected using an Indirect Immunofluorescence assay (IFA). Initially, the sf9 cells were fixed with 4% paraformaldehyde fixative, and subsequently, cells infected with the P3 generation recombinant baculovirus were introduced. To ensure permeability, the cell membranes were treated with TritonX-100. Primary antibodies, specifically a mouse-derived anti-PEDV S1 protein monoclonal antibody (Qianxun Biological, China) and a rabbit-derived anti-TGEV S1 protein polyclonal antibody (Alpha Diagnostic International, USA), were employed. Following this, secondary antibodies were used, including a goat anti-mouse IgG-FITC antibody or a goat anti-rabbit IgG-FITC antibody (Beyotime, China).The resulting images were captured and observed utilizing an inverted fluorescence microscope known as Eclipse Ti-S (Nikon, Japan). These images were then saved for further analysis (41).

### 2.6 Expression time phase analysis

High Five cells can express exogenous proteins more efficiently, so in this study, we analyzed the expression time phase of recombinant proteins in terms of harvesting time and inoculation dose to find out the optimal expression conditions. The steps were as follows: adjust the density of suspended High Five cells to 2×106 cells/mL, inoculate the cells with each recombinant baculovirus at MOI=1, 5, 10, and incubate the cells in suspension at 140 r/min at 27°C, and then take the suspension at 48 h, 72 h, 96 h and 120 h after inoculation to prepare protein samples. The levels of recombinant protein expression were assessed using Western Blot analysis. The grayscale values of the protein bands were quantitatively analyzed with the assistance of ImageJ software. Subsequently, the data obtained from the temporal phase of recombinant protein expression were plotted to visualize the results effectively.

### 2.7 Protein purification and morphology detection

A recombinant baculovirus was introduced into High Five cells to produce a large amount of protein. The cell cultures were harvested and sonicated, and the resulting mixture was centrifuged at 5000 r/min for 50 minutes. The supernatant was then transferred to Millipore ultrafiltration tubes and subjected to further centrifugation. To concentrate the protein samples, ultracentrifugation was performed using sucrose solutions with concentrations of 70%, 50%, and 30%. Finally, the proteins from different protein loops were collected using a syringe. The proteins collected were subsequently filtered through a 0.22 µm membrane to remove impurities. The resulting filtrate underwent SDS-PAGE electrophoresis and was stained with BeyoBlue™ Komas Brilliant Blue Ultrafast Staining Solution to assess VLP purification. The concentration of recombinant proteins was quantified using the Thermo Scientific Pierce™ BCA Protein Assay Kit (Thermo Fisher Scientific, USA) as per the manufacturer’s instructions. The percentage of target proteins was determined by analyzing grayscale values using Image J software.

TEM is a highly effective experimental technique for investigating internal swelling structures in materials. To confirm the self-assembly phenomenon of ADDomer, four proteins were examined using a Talos F200S transmission electron microscope (FEI, USA). The samples were applied onto a carbon-coated grid and negatively stained with a 2% phosphotungstic acid solution for 1 minute. Subsequently, the grid was air-dried for 6 hours and subjected to TEM analysis for observation of the samples’ internal structures.

### 2.8 Vaccine preparation and piglet immunization experiments

Each purified ADDomer-VLP was adjusted to a concentration of 50 µg/mL using sterile PBS. Subsequently, it was emulsified with ISA 201VG adjuvant (Seppic, France) at a 1:1 ratio. This emulsification process was carried out in a biosafety cabinet using a magnetic stirrer to ensure safety. The resulting mixture was prepared and analyzed to assess the physicochemical properties of the vaccine.

Four-week-old castrated male Large White × Duroc binary cross piglets, sourced from a pig farm in Guangdong Province, were selected for the study. Before the experiment, the piglets were subjected to antigen and antibody tests to confirm their negative status for PEDV/TGEV. The experimental animal procedures were approved by the Experimental Animal Ethics Committee of South China Agricultural University (No. 2021F503).

**Figure 1:**
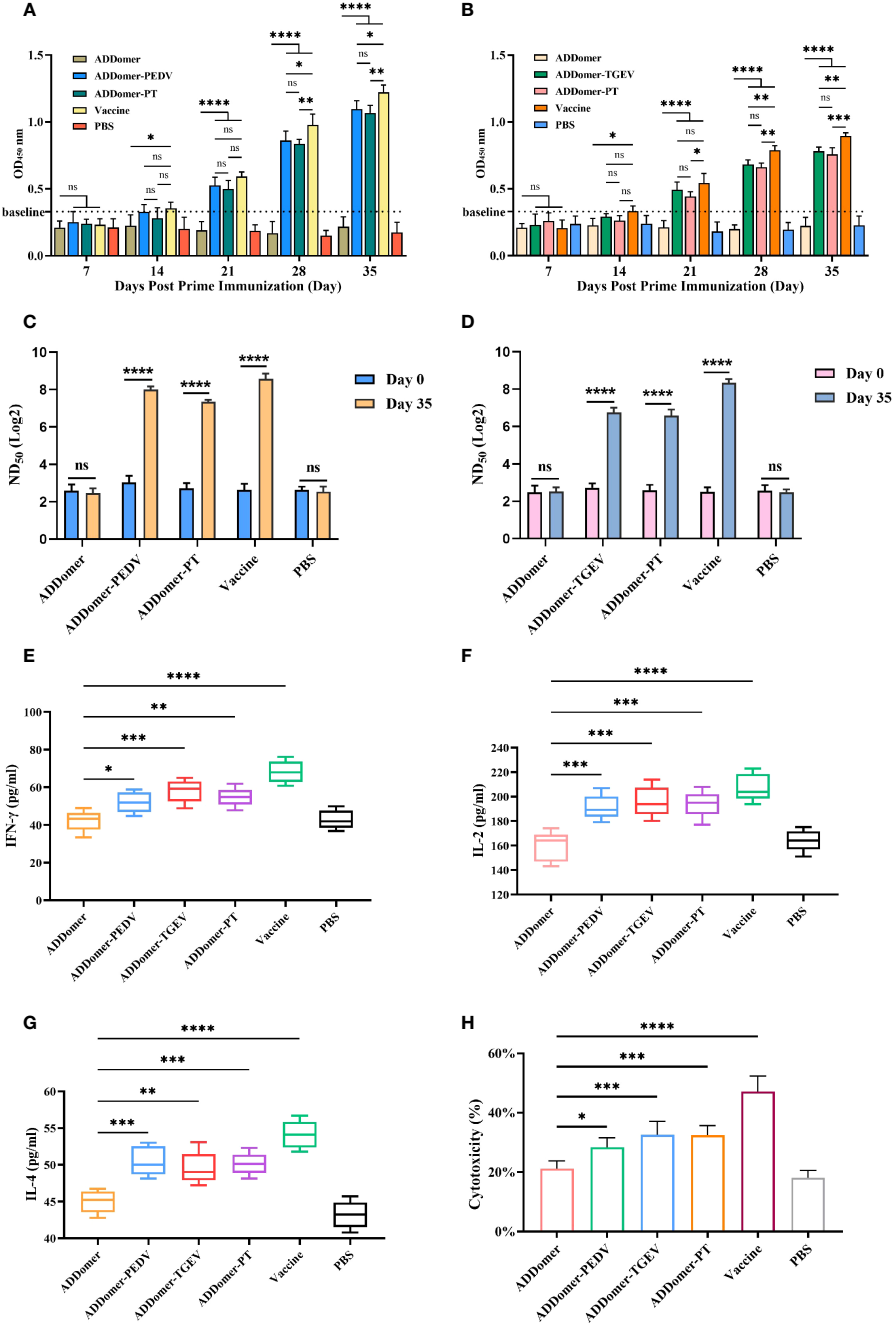
Evaluation of immunization effect in piglets. **(A, B)** Detection of PEDV-specific and TGEV-specific antibodies in the peripheral blood of piglets with commercial kits. **(C, D)** Detection of anti-PEDV and anti-TGEV neutralizing antibodies ND_50_ in peripheral blood of piglets. **(E–H)** Detection of IFN-γ, IL-2, IL-4, and CTL in peripheral blood of piglets at d 35 with commercial kits. *P<0.05, **P<0.01, ***P<0.01, ****P<0.0001, ns >0.05.

The piglets were randomly divided into 6 groups of 3 piglets each. Groups 1-6 were vaccinated with AD vaccine, AD-P vaccine, AD-T vaccine, AD-PT vaccine, PEDV/TGEV weakly virulent vaccine, and PBS immunized control group, respectively. The vaccine formulations used in this study were all W/O/W emulsions, administered via intramuscular injection (**Table 2**). Peripheral blood samples were collected from the piglets to isolate serum, and on day 35, peripheral blood was also used to isolate lymphocytes. The piglets’ body temperature was monitored twice daily at fixed intervals throughout the immunization trial, and their clinical signs were observed. The piglets’ weights were recorded on days 0, 14, and 35 after immunization to calculate the average daily weight gain (**Figure 1A**).

**Table 2:** Piglet immunization program design.

Group	Antigens	Type and composition	Immunization dose and method
1	AD (Negative control)	W/O/W, ISA 201 VG	2 mL (50 µg)/pig;im
2	AD-P	W/O/W, ISA 201 VG	2 mL (50 µg)/pig;im
3	AD-T	W/O/W, ISA 201 VG	2 mL (50 µg)/pig;im
4	AD-PT	W/O/W, ISA 201 VG	2 mL (50 µg)/pig;im
5	PEDV/TGEV diphasic attenuated vaccine	SCJY-1+SCSZ-1	1 mL/pig;im
6	PBS (Non-immune control)	PBS	2 mL/pig;im

### 2.9 The determination of antibody level

The levels of piglet anti-PEDV/TGEV specific antibodies were determined using the porcine PEDV IgG indirect ELISA kit (Ruixin Biotech, China) and the porcine TGEV IgG indirect ELISA kit (Ruixin Biotech, China), following the provided instructions. Peripheral blood samples were collected from piglets on days 7, 14, 21, 28, and 35. The PEDV/TGEV-specific antibody levels were measured in these samples using the respective ELISA kits.

### 2.10 The determination of VNT

VNT(Virus Neutralization Test) assays were conducted to measure the peripheral serum antibody neutralization titers on day 0 and day 35. Vero and ST cells were cultivated in 96-well cell culture plates until a monolayer was formed, ensuring their optimal condition for experimentation. The serum samples collected on day 0 and day 35 were inactivated by exposing them to a 56°C water bath for 30 minutes, and then diluted at an initial ratio of 1:10. Subsequently, the serum was further diluted to a 1:640 ratio at a 1:2 ratio, followed by a 2-hour pre-reaction with 100 TCID_50_ of PEDV/TGEV. After that, the virus/serum mixture was added to the cell wells and incubated for 1 hour. The supernatant was discarded, and serum-free DMEM medium was added to the wells. Eight replicate wells were set up for each group. The cytopathic effects of the cells in each well were observed and recorded. The neutralizing antibody potency (ND_50_) of the peripheral blood from piglets was calculated using the Reed-Muench method.

### 2.11 Cytokine assay

Peripheral blood cytokines in piglets at day 0 and day 35 were analyzed using ELISA kits (MEIMIAN, China). Specifically, the pig IFN-γ ELISA kit, pig IL-2 ELISA kit, and pig IL-4 ELISA kit were employed for this purpose. The levels of IFN-γ, IL-2, and IL-4 in the peripheral blood of each group of piglets were measured following the instructions provided by the respective ELISA kits.

### 2.12 CTL activity detection

To evaluate the CTL activity in peripheral blood, peripheral blood lymphocytes were isolated at day 35. The isolation process was performed using the porcine peripheral blood lymphocyte isolation kit (TBD, China). Subsequently, the CTL activity in piglet serum was determined using the Lactate dehydrogenase (LDH) assay. The LDH cytotoxicity assay kit (Beyotime, China) was employed for this purpose, following the provided instructions.

### 2.13 Statistical analysis

Statistical analysis between groups was performed using GraphPad Prism 9 software. One-way ANOVA and two-way ANOVA were utilized for this purpose. Statistical significance was considered at the following levels: * for *P<*0.05, ** for *P<*0.01, *** for *P<*0.001, and **** for *P<*0.0001.

## 3 Results

### 3.1 Design and structure prediction of recombinant proteins

**Figure 2:**
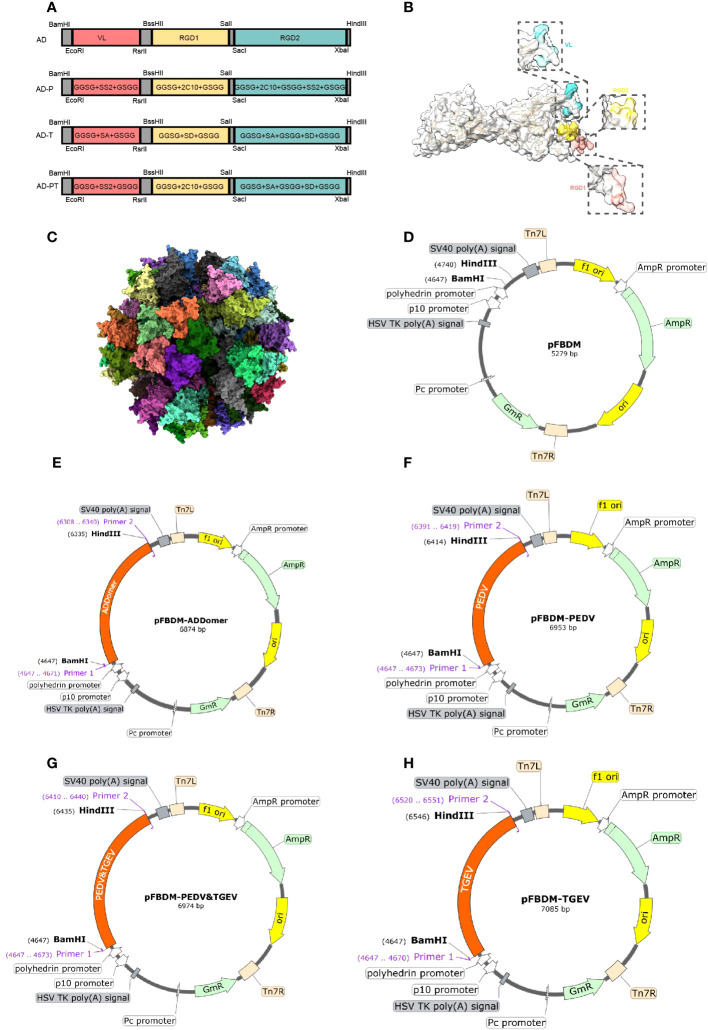
Recombinant protein sequence design. **(A)** The designed sites of recombinant protein on ADDomer, the red region is the VL region in the ADDomer sequence, the yellow region is the RGD1 region, and the green region is the RGD2 region. **(B)** Simulation of the spatial structure of ADDomer protein in the monomeric state, with the VL region in blue, the RGD1 region in red, and the RGD2 region in yellow; **(C)** The synthetic self-assembling ADDomer particle formed by 60 identical protomers. The protomers assemble into 12 pentons, forming a dodecahedron characterized by remarkable thermostability. **(D–H)** Schematic diagram of the recombinant transfer vector, with the designed sequence inserted into the pFBDM vector.

To generate recombinant proteins AD-P, AD-T, and AD-PT, the ADDomer framework was utilized, allowing the insertion of different tandem forms of exogenous antigens (**Figure 2A**). Using UCSF Chimera X, the spatial structure of ADDomer-VLP was simulated, revealing that the VL region, RGD1 region, and RGD2 region of the ADDomer framework are located on the surface of the ADDomer subunit monomer, enabling the carrying of exogenous antigens (**Figure 2B**). The assembled VLP formed a spherical particle consisting of 60 aggregates, facilitating the presentation and delivery of antigens (**Figure 2C**). Through sequence synthesis according to the designed sequences, and subsequent ligation to the transfer vector pFBDM, four recombinant pFBDM plasmids were obtained (**Figures 2D–H**).

### 3.2 Identification and titer determination of baculovirus

**Figure 3:**
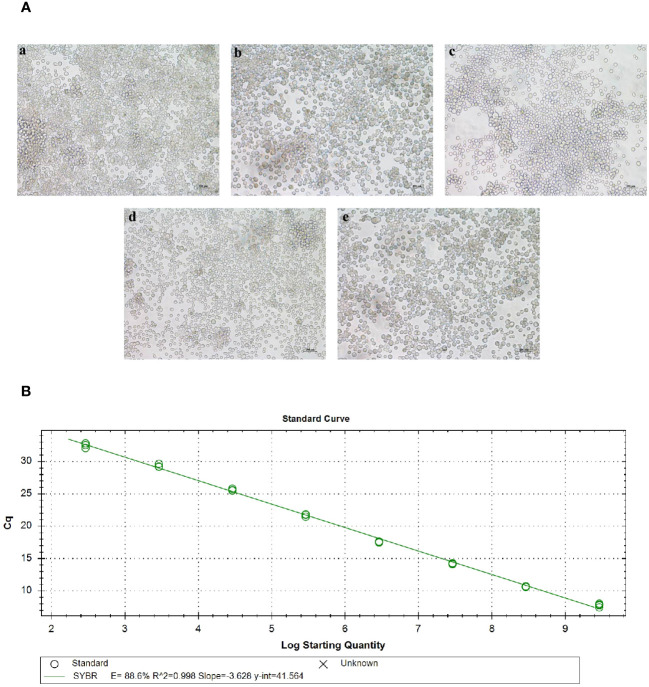
Acquisition of baculovirus. **(A)** Picture of lesions in sf9 cells after baculovirus plasmid transfection. a, b, c, and d represent sf9 cells transfected with rMultibac-ADDomer, rMultibac-ADDomer-PEDV, rMultibac-ADDomer-TGEV, and rMultibac-ADDomer-PT, respectively. e represents normal sf9 cells. **(B)** Mycobacteriophage plasmid copy number standard curve.

The recombinant baculovirus plasmid was successfully constructed and transfected into sf9 cells during their logarithmic growth phase. After 72 hours of transfection, the cells were observed under an inverted microscope. Diseased cells exhibited typical cytopathic effects (CPE), characterized by larger size, swollen nucleus, and significant shedding and cell death. In contrast, normal cells displayed a clear outline, regular morphology, and no shedding or floating phenomenon (**Figure 3A**). The cell culture fluid supernatant was collected to obtain the P1 generation of recombinant baculoviruses, including Ac-AD, Ac-AD-P, Ac-AD-T, and Ac-AD-PT.

The P3 generation of recombinant baculoviruses was assessed for virus titer using absolute quantitative RT-qPCR. The results demonstrated that the constructed baculovirus standard curve exhibited good linearity (**Figure 3B**). By substituting the corresponding Cq values into the standard curve, the copy numbers of P3 generation recombinant baculoviruses were determined. The calculated copy numbers were as follows: Ac-AD (7.31×106 copies/µL), Ac-AD-P (6.46×106 copies/µL), Ac-AD-T (7.64×106 copies/µL), and Ac-AD-PT (2.38×106 copies/µL). The viral titers were subsequently calculated using the formula, resulting in the following values: Ac-AD (1.38×108 pfu/mL), Ac-AD-P (1.25×108 pfu/mL), Ac-AD-T (1.44×10^8^ pfu/mL), and Ac-AD-PT (4.49×10^7^ pfu/mL).

### 3.3 Expression and identification of recombinant proteins

**Figure 4:**
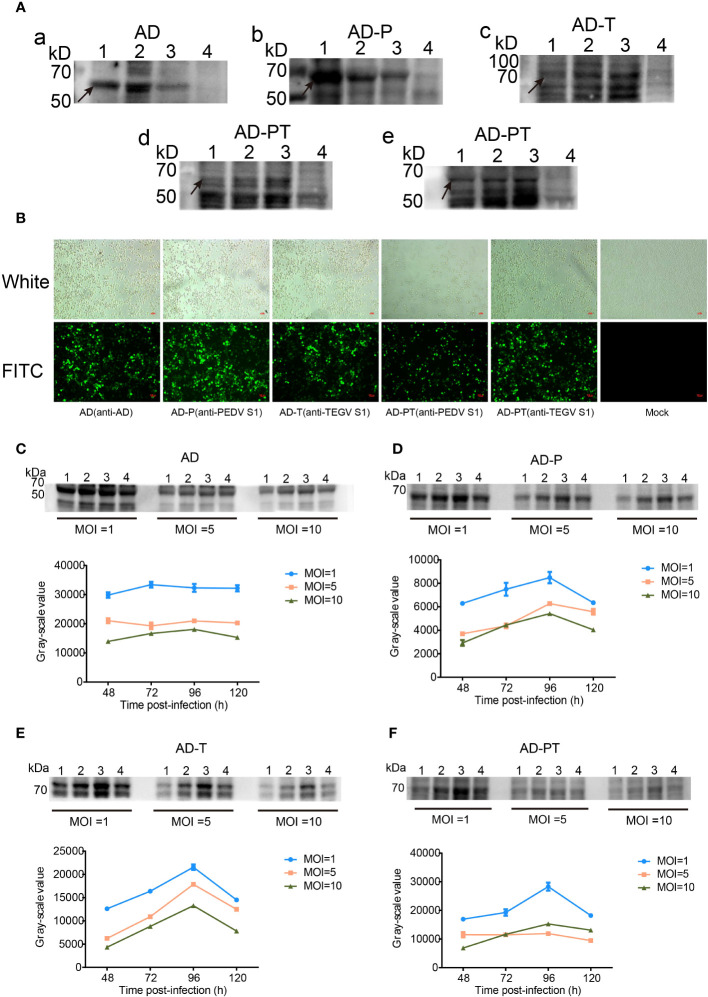
Expression and characterization of target proteins. **(A)** a-e are Western Blot analyses of recombinant proteins. d Primary antibody is mouse-derived anti-PEDV S1 protein monoclonal antibody, e Primary antibody is rabbit-derived anti-TGEV S1 protein polyclonal antibody. 1-3 Lane: P1-P3 generation of recombinant proteins, 4 Lane: negative control. **(B)** Indirect immunofluorescence identification plots of recombinant proteins under white light and fluorescence. **(C-F)** Upper panel shows Western Blot analysis of the changes in expression of recombinant proteins AD, AD-P, AD-T, and AD-PT. 1-4 Lane: Western Blot identification plots of recombinant proteins at 48 h, 72 h, 96 h, and 120 h after recombinant baculovirus infection. The corresponding gray value-time folding plots are shown below.

The expression of recombinant proteins (AD, AD-P, AD-T, and AD-PT) was confirmed through Western Blot analysis (**Figure 4A**). The results revealed the presence of specific bands at approximately 60 kDa, 64 kDa, 70 kDa, and 69 kDa, respectively, corresponding to the recombinant proteins. However, it is worth noting that there were additional heterobands observed near the target bands, which could be attributed to the depolymerization of multimeric proteins by SDS and the nonspecific binding of other heteroproteins to the primary antibody in the samples.

Additionally, the expression of recombinant proteins from the P3 generation of recombinant baculoviruses was assessed using IFA (**Figure 4B**). The sf9 cells infected with the recombinant baculovirus exhibited specific green fluorescence, indicating the successful construction of the recombinant baculovirus and the expression of the recombinant proteins in sf9 cells with good reactogenicity.

The expression of recombinant proteins was enhanced through optimization of time and MOI (**Figures 4C–F**). Among the different variants, namely AD protein, AD-P protein, AD-T protein, and AD-PT protein, the highest protein expression was observed when the MOI was set to 1 and the infection duration was 96 hours. Consequently, an MOI of 1 was chosen as the optimal infection dose, and an infection duration of 96 hours was deemed optimal for viral amplification and collection of the recombinant protein solution.

### 3.4 Purification of recombinant protein and TEM observation

**Figure 5:**
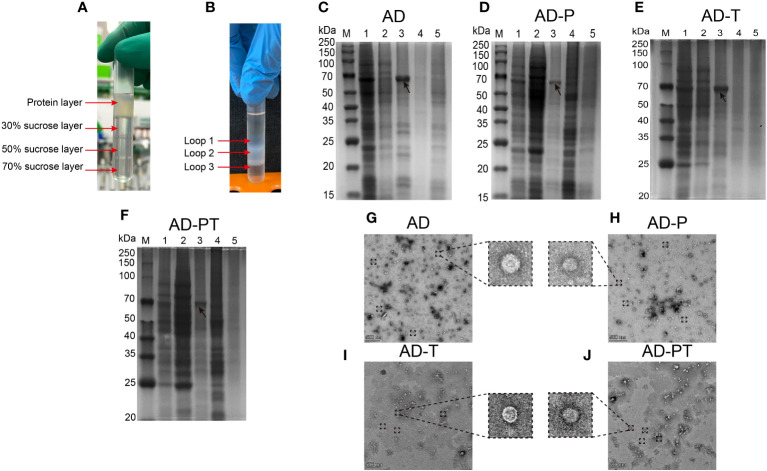
Purification of recombinant proteins and TEM table evidence **(A)** Protein samples before purification by sucrose gradient. **(B)** Distribution of each recombinant protein sample in the ultrafiltration tube after sucrose gradient centrifugation. **(C–F)** The results of the recombinant protein staining with Thomas Brilliant Blue, Lane M: Protein Marker; Lane 1: Protein sample before sucrose gradient purification; Lane 2: Mezzanine 1 protein sample; Lane 3: Mezzanine 2 protein sample; Lane 4: Mezzanine 3 protein sample; Lane 5: Negative control; **(G-J)** Recombinant ADDomer-VLP under transmission electron microscope 14000x field of view, the zoom field of view of individual VLPs is shown in the large dashed box.

The recombinant protein was added to the ultracentrifuge tube and floated above the sucrose (**Figure 5A**). Following the centrifugation of the recombinant proteins using a sucrose density gradient, distinct “protein loops” were observed in the centrifuge tube (**Figure 5B**). These protein loops were most prominent within the sucrose concentration range of 30%-50%. Subsequently, the protein ring samples were subjected to SDS-PAGE analysis. The purified products of each recombinant protein exhibited specific bands at their expected positions, with fewer peripheral bands indicating reduced heterogeneity (**Figures 5C–F**). This observation suggested that the majority of the purified recombinant proteins were concentrated within the 30%-50% sucrose concentration range. The final concentrations of the purified recombinant proteins, AD, AD-P, AD-T, and AD-PT, were determined as 202 µg/mL, 110 µg/mL, 83 µg/mL, and 87 µg/mL, respectively. These concentrations were calculated using the BCA assay and grayscale value analysis.

The purified VLPs, as observed by TEM, exhibited diameters ranging approximately from 20 to 40 nm. However, the overall field of view exhibited high impurities, including many sucrose crystals remaining (**Figures 5G–J**). These findings confirm the successful purification of the respective recombinant proteins, which demonstrated their ability to self-assemble *in vitro* and form VLPs utilizing ADDomer as the structural framework. These VLPs can now be employed as immunogens for the upcoming piglet immunization study.

### 3.5 Clinical manifestations

**Figure 6:**
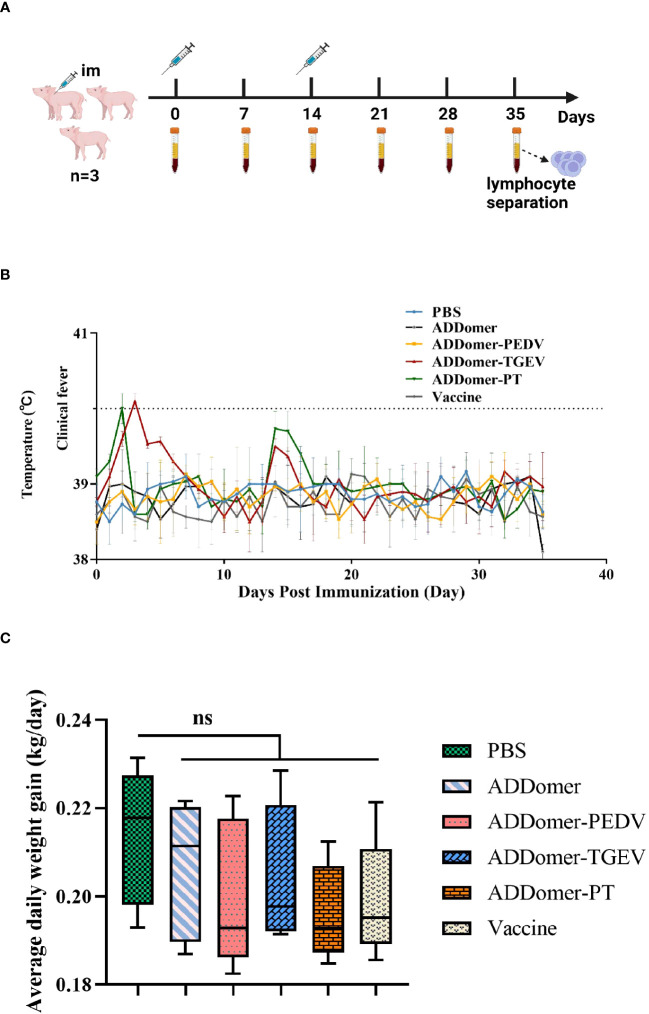
Immunization schedule and body temperature and weight changes in piglets **(A)** Immunization trial design for piglets with intramuscular injections and prior blood collection at weeks 0 and 2, respectively. The negative control group was piglets injected with PBS. **(B)** Body temperature changes of piglets during the immunization trial. **(C)** Statistical results of average daily weight gain of piglets. Note: ns represents no statistically significant difference between the current experimental group and the PBS group (*P*>0.05).

The piglets underwent immunization according to the designated plan (**Figure 6A**). Following a 35-day immunization period, all piglets survived. Additionally, they displayed a positive demeanor and showed no signs of inflammation or swelling at the immunization site. However, piglets in the AD-P group experienced a temporary loss of appetite on the 7th day post-immunization. This symptom swiftly improved upon administering a combination of multivitamin powder and Astragalus polysaccharide powder in their drinking water. In contrast, the piglets in the remaining experimental groups did not manifest any discernible clinical symptoms.

### 3.6 Body temperature change

Rectal temperatures of the piglets were monitored in all experimental groups (**Figure 6B**). Except for the AD-T and AD-PT immunized groups, no notable instances of fever (body temperature ≥40°C) were detected at any time point. The AD-T group experienced a slight elevation in body temperature on the 3rd day after immunization, while the AD-PT group exhibited a similar increase on the 2nd day post-immunization.

### 3.7 Weight change

Each piglet’s weight was measured before vaccination as well as on days 14 and 35 afterward. The piglets’ average daily weight gain was computed and statistically examined (**Figure 6C**). The findings showed that there were no differences in the mean daily weight gain between any of the immunized groups and the PBS-treated non-immunized control group (*P*>0.05). It is crucial to recognize that variations in piglet body weight can be influenced by a variety of elements, including the feeding environment and feed palatability. Therefore, the observed differences in body weight across the various groups may have been caused by these causes.

### 3.8 Specific antibody testing in peripheral blood

We measured the levels of PEDV and TGEV antibodies in piglets’ peripheral blood at days 7, 14, 21, 28, and 35 using an indirect ELISA kit. The statistical results were expressed as OD values measured at 450 nm. Samples were considered positive for PEDV and TGEV when the OD values exceeded baseline values of 0.33 and 0.34, respectively (**Figures 1A, B**).

Comparison of PEDV-specific antibody levels at day 35 revealed the following: The AD-P vaccine immunization group and the AD-PT vaccine immunization group did not show a significant difference (*P*>0.05). However, there was a significant difference between the commercialized attenuated vaccine immunization group and the AD-P vaccine immunization group (*P*<0.05) and compared to the AD-PT vaccine immunization group (*P*<0.01). There was also a significant difference between the AD vaccine-immunized group and the other vaccine-immunized groups (*P*<0.0001). These results indicate that both AD-P and AD-PT based on baculovirus expression system have good immunogenicity and can induce the body to generate humoral immune response against PEDV.

Comparison of TGEV-specific antibody levels at day 35 revealed the following: There was a significant difference (*P*<0.01) between the group immunized with the commercial weakened vaccine and the group immunized with the AD-T vaccine and a significant difference (*P*<0.001) compared to the group immunized with the AD-PT vaccine. There was no significant difference between the AD-T and AD-PT vaccine immunization groups (*P*>0.05). There was a significant difference between the AD vaccine-immunized group and the other vaccine-immunized groups (*P*<0.0001). These results indicate that both AD-P and AD-PT based on baculovirus expression system have good immunogenicity and can induce the body to generate humoral immune response against TEGV.

### 3.9 Peripheral blood-neutralizing antibody test

To verify the protective effect induced by recombinant ADDomer-VLP in piglets, serum micro-neutralization experiments were performed *in vitro*. We determined the changes in ND50 of serum against PEDV and TGEV in piglets at day 0 and day 35 after immunization, respectively (**Figures 1C, D**).

There was no significant difference in the changes of anti-PEDV neutralizing antibodies between piglets in the AD vaccine-immunized and PBS groups before and after immunization (*P*>0.05). However, the ND50 of the sera of piglets in the AD-P, AD-PT and commercial weak vaccine immunized groups on the 35th d were elevated and significantly different (*P*<0.0001) from those before immunization. Their neutralizing antibody titers reached 258, 157 and 445, respectively. The above results indicate that AD-P and AD-PT vaccines induced different degrees of protection against PEDV in the organism.

There was no significant difference in the changes of anti-TGEV neutralizing antibodies between the AD vaccine-immunized and PBS groups of piglets before and after immunization (*P*>0.05). However, the ND50 of serum of piglets in the AD-T, AD-PT and commercial weak vaccine immunized groups on the 35th d were all elevated and significantly different from those before immunization (*P*<0.0001). Their neutralizing antibody titers reached 119, 97and 315, respectively. The above results indicate that AD-T and AD-PT vaccines induced different degrees of protection against TGEV in the organism.

### 3.10 Peripheral blood IFN-γ, IL-2, and IL-4 assays

We employed an indirect ELISA kit to measure IFN-γ and IL-2 concentrations in peripheral blood samples from piglets on day 35 to assess the Th1-type immune response level (**Figures 1E, F**).

There were significant differences in serum IFN-γ concentrations in piglets from AD-P and AD-PT vaccine-immunized groups compared with AD vaccine-immunized groups (*P*<0.05). Among them, IFN-γ concentration was higher in the AD-T vaccine-immunized group than in the AD-P and AD-PT vaccine-immunized groups. However, all of them had lower IFN-γ concentrations than the immunized group with commercial weak vaccine. The above results indicate that all the recombinant ADDomer-VLP vaccines prepared in this study were able to induce an increase in the concentration of IFN-γ in the organism, generate a Th1-type immune response, and trigger cellular immunity. There were significant differences in piglet serum IL-2 concentrations from the AD-P, AD-T, and AD-PT vaccine-immunized groups compared with the AD vaccine-immunized group (*P*<0.001). The above results indicated that all recombinant ADDomer-VLP vaccines prepared in this study were able to induce an increase in the concentration of IL-2 in the organism, generate a Th1-type immune response, and trigger cellular immunity.

To assess the level of Th2 immune response in the peripheral blood of piglets after immunization, the concentration of IL-4 in the peripheral blood at day 35 was measured using an indirect ELISA assay kit (**Figure 1G**). There were significant differences in IL-4 concentration in serum of piglets in the AD-P, AD-T and AD-PT vaccine-immunized groups compared with the PBS group (*P*<0.01). Among them, IL-4 concentrations were higher in the AD-P and AD-PT vaccine-immunized groups than in the AD-T vaccine-immunized group. The above results indicate that all recombinant ADDomer-VLP vaccines prepared in this study are capable of inducing an increase in the concentration of IL-4 in the organism, generating a Th2-type immune response and triggering a high level of humoral immunity.

### 3.11 Peripheral blood CTL activity test

LDH can be used as an indicator of cytotoxicity because it is steadily released from the cytoplasm when cellular structures are damaged. Therefore, we measured CTL activity in the peripheral blood of piglets on the 35th day by the LDH method to evaluate the level of cellular immunity in the body (**Figure 1H**). There were significant differences in CTL activity in the other immunized groups compared to the AD vaccine-immunized group (*P*<0.05). Among them, the peripheral blood CTL activities of piglets in the AD-P, AD-T and AD-PT vaccine-immunized groups were about 30%, 35% and 33%, respectively. Moreover, the peripheral blood CTL activity of piglets in the commercial vaccine group reached 47%. The above results indicate that all recombinant VLPs based on baculovirus expression system induced higher CTL activity in piglets.

## 4 Discussion

Since 2019, COVID-19 has exerted significant global pressure on public health, leading to increasing demand for coronavirus prevention and control measures (42). Within the pig breeding industry in China, SeCoVs such as PEDV and TGEV have inflicted substantial losses and persist as ongoing challenges. The emergence of PDCoV, another SeCoV, with potential transmission to humans, has raised concerns and alerted us to the potential dangers of SeCoVs (43). These two viruses commonly manifest as mixed infections clinically and exhibit synergistic effects with other enterovirus infections (44). Vaccination is the primary method for the prevention and control of PEDV and TGEV. However, due to the rapid mutation and frequent recombination of both strains, traditional vaccines derived from specific strains are insufficient in providing effective protection. Traditional attenuated vaccines carry the potential risk of viral spread, whereas inactivated vaccines are less effective and necessitate multiple immunizations with large doses (45). Hence, there is a pressing need to explore novel genetically engineered vaccines that offer improved safety and efficacy for the prevention and control of PEDV and TGEV.

A study reported that expression of a conserved epitope of influenza virus in Salmonella flagellin produced effective protection in mice (46). Most of the studies were conducted through immunoinformatics analyses, which involved studying dominant epitopes, designing epitope vaccines, establishing structural models, and conducting immune simulation analysis. These investigations served as preliminary studies on epitope vaccines, but their findings still require validation through experimental methods (47–51). We selected four neutralizing antigenic epitope regions in the S proteins of PEDV and TGEV, respectively, based on the results obtained experimentally by our predecessors. SS2 and 2C10 induce PEDV-specific neutralizing antibodies in mice (32, 33). The A and D sites induce the body to produce neutralizing antibodies against TGEV (52, 53). In our study, we selected the highly conserved SS2 and 2C10 as the target antigen epitope region for the PEDV S protein. Similarly, for the TGEV S protein, we chose site A and site D as the target antigen epitope region. These selections were made to prepare the PEDV/TGEV VLP vaccine.

ADDomer has been used in the development of vaccines for human and animal diseases (23, 24, 40, 54, 55). BEVS, known for its low cost and ability to facilitate post-translational protein modifications, is the preferred method for preparing VLP vaccines in actual production. The combination of ADDomer and BEVS in producing VLP vaccines not only exhibited superior epitopes but also demonstrated excellent immunogenicity (40).

We utilized BEVS to obtain recombinant proteins AD, AD-P, AD-T, and AD-PT. After sucrose gradient centrifugation purification, we used TEM to observe the assembly morphology of the recombinant VLPs. The results revealed that the VLPs exhibited similar morphologies to natural viral particles, indicating the successful preparation of recombinant VLPs carrying PEDV and TGEV antigenic epitopes. Following immunization in piglets, specific antibodies, neutralizing antibodies, IFN-γ, IL-2, and IL-4 levels were elevated in the piglets’ bodies. Moreover, the immunization induced higher CTL activity, triggering both PEDV/TGEV-specific humoral and cell-mediated immune responses.

To preliminarily investigate the immunogenicity of the recombinant ADDomer-VLP, this study prepared the purified recombinant ADDomer-VLP and ISA 201VG adjuvant in a 1:1 emulsion to formulate the vaccine. The immune response of the ADDomer-VLP vaccine was evaluated by immunizing 4-week-old piglets. Following immunization, no significant adverse reactions were observed in any of the vaccinated groups, and the vaccine did not adversely affect the growth status of the piglets, demonstrating that the prepared ADDomer-VLP vaccine exhibits excellent safety.

IgG is the primary antibody produced by the body and serves as a key antibody in serological diagnosis and post-immunization detection. During the immune response, IgG plays a role in complement activation and neutralization of various toxins. IgG antibodies not only have a long duration of action but are also the only antibodies that can cross the placenta to protect the fetus. IgG can be transferred to newborns through colostrum, which is crucial for their protection against infections. The AD-P vaccine induces a slightly lower level of anti-PEDV antibodies in the body compared to commercial attenuated vaccines but slightly higher than the AD-PT vaccine. The AD-T vaccine stimulates the production of TGEV-specific antibodies at a level slightly lower than commercial attenuated vaccines but slightly higher than the AD-PT vaccine. The AD-PT vaccine can simultaneously stimulate the production of specific antibodies against both PEDV and TGEV. It is speculated that the results may be attributed to the higher density and specificity of the PEDV/TGEV neutralizing antigenic epitopes displayed on the surface of AD-P and AD-T compared to AD-PT. All vaccines induce piglets to produce high levels of specific antibodies against PEDV and TGEV, triggering a robust humoral immune response.

One of the most crucial indicators for evaluating vaccine effectiveness is the titer of neutralizing antibodies in the body. Neutralizing antibodies can block viral infections by binding to the virus and causing spatial hindrance effects. Thus, the titer of neutralizing antibodies in the blood is essential as it directly reflects the immune protective effect. In this study, piglet peripheral blood serum samples were collected on day 0 and day 35, and the changes in neutralizing antibody levels against 100 TCID_50_ of PEDV and TGEV were examined before and after immunization. The results showed that the serum from the AD-P vaccine group had an ND_50_ of 258 against PEDV, which was superior to the AD-PT vaccine group (ND_50 =_ 157). Additionally, the serum from the AD-T vaccine group had an ND_50_ of 119 against TGEV, outperforming the AD-PT vaccine group (ND_50 =_ 97). Except for the AD vaccine group, the serum from all the recombinant ADDomer-VLP vaccine groups exhibited extremely significant differences (*P*<0.0001) in ND50 on day 35 compared to day 0. While both AD-T and AD-PT vaccines demonstrated good immune effects, their neutralizing antibody titers were slightly lower. This could be attributed to the relatively complex conformation of the TGEV S protein antigen sites after translation, resulting in suboptimal exposure levels and spatial configurations of sites A and D. Consequently, this may have affected their ability to stimulate the host’s immune response. The serum from the AD-P vaccine group displayed a stronger neutralizing antibody titer, although it did not reach the level of commercial bivalent attenuated vaccines. In conclusion, the SS2 and 2C10 epitopes can still serve as potential candidate antigenic sites for novel PEDV vaccines.

After vaccination, the levels of IFN-γ and IL-4 in the body reflect Th1 and Th2 cell responses, respectively. Typically, Th1 immune responses are pro-inflammatory and can trigger cell-mediated immune reactions, while Th2 immune responses are anti-inflammatory and lead to humoral immune responses. IL-2 is mainly synthesized by CD4+ T cells upon antigen stimulation, promoting T cell proliferation and inducing CTL responses by acting on CD8+ T cells. Therefore, IFN-γ/IL-2 and IL-4 are representative indicators used to assess the degree of cell-mediated and humoral immune responses triggered by vaccines. In this study, cytokine analysis was performed on the peripheral blood serum of piglets on day 35. The results showed that AD-T and AD-PT vaccines had a more significant effect on elevating IFN-γ levels in piglets compared to the AD-P vaccine. Conversely, the AD-P vaccine showed a more pronounced increase in IL-4 levels compared to the AD-T and AD-PT vaccines. This suggests that the SS2 and 2C10 epitopes of PEDV tend to induce humoral immune responses, while the A and D epitopes of TGEV lean towards inducing cell-mediated immune responses. Moreover, the AD-PT vaccine, which carries all four epitopes mentioned above, can simultaneously stimulate the production of both humoral and cell-mediated immune responses in the host.

CTL is the primary effector cells in cell-mediated immune responses. It exerts their immune functions by secreting various cytokines and plays a crucial role in clearing viral infections within the body. The results of the CTL activity assay conducted on day 35 in the peripheral blood of piglets revealed significant differences (*P*<0.05) in CTL activity between all the groups immunized with ADDomer-VLP vaccines carrying exogenous antigenic epitopes and the AD vaccine group. This indicates that each recombinant ADDomer-VLP vaccine can induce a cellular immune response in the body. Among them, the AD-T vaccine induced higher CTL activity compared to the AD-P and AD-PT vaccines, which carry the SS2 and 2C10 antigenic epitopes, respectively.

After sucrose gradient centrifugation for the purification of recombinant VLPs, the SDS-PAGE results showed a significant reduction in impurities in the lanes corresponding to 30% to 50% sucrose concentration, indicating the relatively high purity of ADDomer-VLPs at this concentration. However, when observing the structural morphology of ADDomer-VLPs under TEM electron microscopy, there were still many impurities within the field of view, including a large amount of residual sucrose crystallization. This suggests that the purification process of VLPs needs further optimization and improvement. In the actual production process, the preparation of VLPs, especially downstream processing, faces significant challenges such as low yield, lack of platform-based processes, and rapid analytical techniques (56). Ultracentrifugation is commonly used for purification at the laboratory scale, but it can lead to issues such as variability in results between different batches. Therefore, the optimization of VLP purification processes remains a focal point in the research and development of VLP vaccines.

For chimeric VLP vaccines, the antigenic epitope display density and insertion method are crucial factors that influence their immunogenicity. The differences in immunization efficacy observed between AD-P, AD-T, and AD-PT groups are attributed to variations in the insertion positions and linkage methods of the antigenic epitopes within the recombinant ADDomer-VLPs. Therefore, future research can explore different insertion positions and concatenation strategies for VLP groups to screen for more effective vaccine design strategies. PEDV and TGEV are intestinal coronaviruses, and mucosal immunity serves as the first line of defense, representing a more direct and efficient form of immune response. Therefore, the detection priority for antibodies should be given to IgA over IgG. The neutralizing titer of peripheral blood serum antibodies in piglets partially reflects the humoral immune level in the body. However, the primary evaluation parameter after vaccination is the vaccine’s protective efficacy against challenge infections. Therefore, future studies should focus on investigating the recombinant ADDomer-VLP vaccine’s protective ability against PEDV/TGEV of different genotypes to assess its broad-spectrum protection. The recombinant ADDomer-VLP vaccine prepared in this study can induce specific humoral and cellular immune responses against PEDV/TGEV. Since newborn piglets primarily rely on maternal antibodies for protection against infection, it is essential to consider applying the research findings to firstborn sows and sows in reserve to evaluate the vaccine’s immunogenicity and protective efficacy in newborn piglets.

In addition, compared to commercialized vaccines, the immune efficacy of the recombinant ADDomer-VLP vaccine we constructed is not as good. Traditional live attenuated vaccines and inactivated vaccines have complete viral particles, which confer good immunogenicity and can activate the body to produce a robust immune response. Our recombinant vaccine expresses dominant antigenic epitopes of the virus, but its immunogenicity is relatively low. Recombinant vaccines require adjuvants or fusion with immune enhancers to improve their immunogenicity. Therefore, we chose the ISA 201VG adjuvant to enhance immunogenicity, but the immune response is still not as effective as that of traditional live attenuated and inactivated vaccines. As we further explore and research dominant antigenic epitopes and adjuvants, epitope-based vaccines hold the potential to replace traditional live attenuated and inactivated vaccines.

In summary, our team has successfully developed an ADDomer-VLP delivery system that carries antigenic epitopes of PEDV/TGEV, resulting in an effective vaccine capable of stimulating both Th1-type and Th2-type immune responses in piglets against PEDV and TGEV infections. This research highlights the potential of ADDomer-VLP as a highly efficient delivery system for PEDV and TGEV epitopes, emphasizing the promising role of the recombinant ADDomer-VLP vaccine in combating PEDV and TGEV infections.

## Data availability statement

The original contributions presented in the study are included in the article/supplementary material, further inquiries can be directed to the corresponding author.

## Ethics statement

The animal study was approved by The Experimental Animal Ethics Committee of South China Agricultural University (No. 2021F503). The study was conducted in accordance with the local legislation and institutional requirements.

## Author contributions

SF, MZ, and JC conceived and designed the research. PD, QY, and WZ carried out the experiment. PD wrote the main manuscript text. KX, ZY, XDL, XYL, LZ, KW, and XWL prepared **Figures 2**–**6**. All authors contributed to the article and approved the submitted version.
